# Crystal Structure Studies of Human Dental Apatite as a Function of Age

**DOI:** 10.1155/2009/698547

**Published:** 2009-06-16

**Authors:** Th. Leventouri, A. Antonakos, A. Kyriacou, R. Venturelli, E. Liarokapis, V. Perdikatsis

**Affiliations:** ^1^Department of Physics and Center for Biological and Materials Physics, Florida Atlantic University, Boca Raton, FL 33431, USA; ^2^Department of Physics, National Technical University of Athens, 15780 Athens, Greece; ^3^Department of Mineral Resources Engineering, Technical University of Crete, 73100 Chania, Greece

## Abstract

Studies of the average crystal structure properties of human dental apatite as a function of age in the range of 5–87 years are reported. The crystallinity of the dental hydroxyapatite decreases with the age. The a-lattice constant that is associated with the carbonate content in carbonate apatite decreases with age in a systematic way, whereas the c-lattice constant does not change significantly. Thermogravimetric measurements demonstrate an increase of the carbonate content with the age. FTIR spectroscopy reveals both B and A-type carbonate substitutions with the B-type greater than the A-type substitution by a factor up to ~5. An increase of the carbonate content as a function of age can be deduced from the ratio of the *ν*_2_CO_3_ to the *ν*_1_PO_4_ IR modes.

## 1. Introduction

Hydroxyapatite (HAp) with multiple substitutions at all sites and containing ~4% to 6% carbonate is the primary component of bones (70% wt) and teeth (96% wt) [1, 2]. Several authors have reported on the structure and properties of human dental carbonate HAp [3–5]. They study the enamel part of the tooth with a focus on the crystallographic structure and the carbonate substitution because carbonate affects important properties of the physiological HAp such as reactivity and solubility [6]. Studies on the graded nature and texture of dental enamel by comparison of the microstructures of slices of human adult and baby canine enamel have been reported [7, 8]. Human deciduous and permanent enamel samples were studied by Fourier transform infrared (FTIR) spectroscopy to determine quantitatively the B-type (carbonate for phosphate) and A-type (carbonate for hydroxyl) carbonate contents in human enamel [9]. The mineral content, crystallite size, and mechanical properties of aging (transparent) human dentin were compared with the ones in normal human dentin in recent studies [10, 11].

We study the average crystal structure properties in bulk human dental apatite as a function of age in the range 5–87 years without separating the enamel from the dentin. We have undertaken a research project that requires a large and diverse origin of specimens in order to correlate the average crystal structure properties of aging dental apatite with the parameters that create the structural changes. Understanding the fundamental science of the dental mineral phase as a function of age could be helpful in efforts of remineralization of human dental apatite [12]. Here we report some preliminary results on systematic trends of average crystal structure parameters and carbonate content in bulk human dental apatite as a function of age by using powder X-ray diffraction (XRD), thermogravimetric analysis (TGA), and FTIR spectroscopy.

## 2. Experimental

Two local dental offices provided teeth samples for this research with the informed consent of their patients. The teeth-samples were selected in the age range 17–87 years old. One healthy 5 year old deciduous tooth was offered by a family member of the first author. The cleanest pieces of each tooth (free of fillings, etc.) were selected under magnification, powdered with an agate mortar and pestle, and passed through a 125 *μ* sieve. The way of preparation of the samples implies that the average crystal structure properties of the dental apatite are studied. Each specimen was labeled with a capital T followed by a number that represents the age of the donor of the tooth.

Powder X-ray diffraction measurements were performed using a Siemens D5000 powder diffractometer operating at 45 kV and 40 mA with Cu-K*α* radiation and a diffracted beam monochromator. Data were collected in the 2*θ* range of 8°–90° with a step size of 0.02° and a counting time of 20 seconds at each step. The data bank from the International Center for Diffraction Data (ICDD) was used in a search/match program for phase identification. The Rietveld refinement method [13] in the GSAS program [14] was used for crystal structure analysis of the diffraction patterns only for the HAp phase. The crystallographic model used space group P63/m with isotropic atomic displacement parameters [4]. First, the scale factor, background, peak profile (pseudovoight function) and lattice parameters were simultaneously refined; then, the atomic positions and isotropic displacement parameters were refined for all the atoms except for the oxygen and hydrogen of the hydroxyl site in the diffraction patterns from the older age teeth, because combination of low occupancy at the channel site, poor crystallization and peak overlapping would create instability of the refinement. For the same reason, the fractions of the Ca1, Ca2, and P atoms were refined in diffraction patterns up to 45-year-old teeth.

Thermogravimetric analysis was performed to evaluate the carbonate content in the samples. The loss of weight as a function of temperature from samples of 50–100 mg was recorded using a PerkinElmer thermogravimetric analyzer TGA-6. The heating rate was 10°C/min in the temperature range 25°C to 950°C. 

FTIR spectroscopy was used to study the carbonate presence in the HAp structure of the specimens. The FTIR spectra were recorded on a Bruker Optic IFS66v/S interferometer equipped with an attenuated total reflectance (ATR) unit. The ATR unit permits the spectra collection without any special sample preparation and it is used for characterization and quantitative estimations in several materials. At a minimum two samples from each age were measured. The range of frequencies was 500 to 4000 cm^−1^ and the spectra were recorded in ambient conditions with a resolution of 2 cm^−1^. In order to obtain a good signal-to-noise ratio, more than a hundred scans were collected and averaged. An KBr beamsplitter was used for the M-IR source.

## 3. Results and Discussion

### 3.1. Powder X-Ray Diffraction

**Figure 1:**
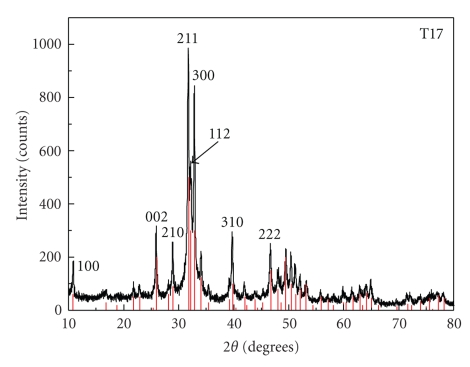
X-ray diffraction pattern of the sample from the 17-year-old donor (T17). All the peaks match the HAp pattern number 9-432 from the ICDS. A few minor peaks of unidentified phases are within detection limits of the method.

Figure 1 shows the X-ray diffraction pattern collected from the sample T17 in comparison with the HAp phase PDF number 9-432. While this is the main identified phase in the diffraction patterns of all samples, minor unidentified phase(s) exist even in the young-age teeth, although within the detection limits of the method in the later. The identified secondary phases vary with the tooth-age qualitatively and quantitatively, as deduced from the Bragg peaks and their relative intensities. Poor crystallinity, broadening, and overlapping of the diffraction peaks would make the phase identification ambiguous especially in old age teeth-samples. Possible secondary phases include the biologically relevant calcium compounds: Ca_2_(P_4_O_12_).4H_2_O (no. 50-582), Ca_8_H_2_ (PO_4_)_6_5H_2_O (no. 26-1056), Ca_6_(CO_2.65_)_2_(OH_.657_)*·*7(H_2_O) (no. 78-1540), CaCO_3_ (no. 71-2392) and Ca_2_P_2_O_7_ (73-440) [6, 15]. Note that the type and number of the secondary phases vary in each specimen.

**Figure 2:**
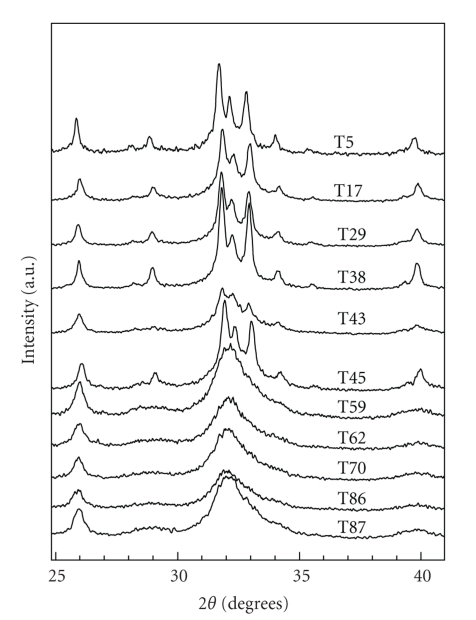
Parts of the XRD patterns displaying the development of the HAp phase in human dental apatite as a function of age.

The X-ray diffraction patterns of Figure 2 reveal a systematic decrease of the crystallinity of human dental apatite from 5–87 years old. It is quite noticeable that patterns collected from teeth up to 45 years old show highly crystallized materials (with the exception of T43), whereas the patterns from older-age teeth display an increasing broadening of the Bragg peaks that indicates an increasing loss of crystallinity of the human dental apatite as a function of age. As it has been demonstrated [6], acids produced by plaque bacteria, acidic food, or drink cause a partial dissolution of dental apatite. Then it is reasonable to expect that aging of dental apatite favors such dissolution which is followed by decrease of the crystallite size. This decrease as a function of age is demonstrated by the broadening of the diffraction peaks of Figure 2. The average crystallite size *τ* was calculated from the FWHM *β* of the (002), and (310) Bragg peaks using the Scherrer formula *τ* = *K λ*/*β* cos  *θ*. These two peaks were chosen because they do not overlap with others. It was found that the average crystallite-size in the specimens varies from ~12 nm (older age teeth) up to ~38 nm (younger age teeth). These numbers are in agreement with values found for crystallites in dentin [10] and enamel [16].

**Figure 3:**
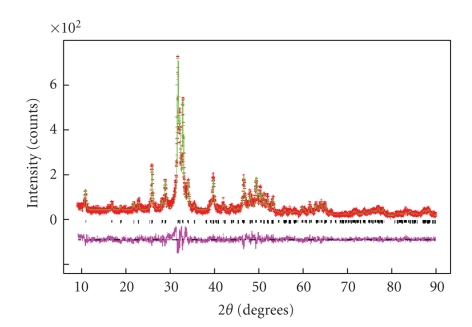
Rietveld refinement of the diffraction pattern collected from the sample T38. Crosses mark the experimental data, the continuous line is the calculated HAp pattern, the vertical ticks mark the calculated Bragg peaks, and the lower trace shows the difference between observed and calculated patterns.

The average crystallographic properties of the specimens were found from Rietveld refinement of the powder diffraction patterns. One example is shown in Figure 3 from the sample T38. The weighted *R* factors of the refined patterns of all the samples were 0.15 ≤ *R*_wp_ ≤ 0.18 except for the T87 with *R*_wp_ = 0.28. The reduced *χ* ^2^ were 1.2 ≤ *χ* ^2^ ≤ 1.6 and the *R*_Bragg_ were 0.06 ≤ *R*_Bragg_ ≤ 0.13. The low counting rate, presence of secondary phases, and nanoscale crystallite size explain such high residuals combined with low goodness of fit. The occupancies of the Ca and P sites refine to values less than one as expected from the chemical composition of dental apatite [1, 2].

**Figure 4:**
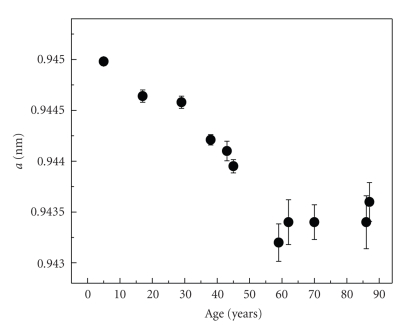
The a-lattice constant of the HAp phase as a function of age. Notice the increase of the error in poorly crystallized dental apatite (older-age teeth).

**Figure 5:**
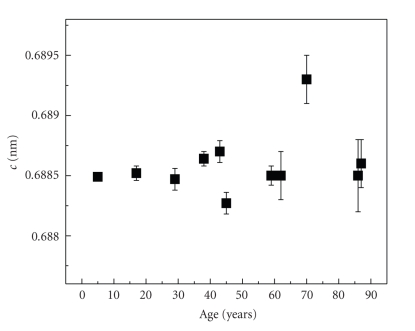
The c-lattice constant as a function of age.

A systematic decrease of the a-lattice constant with the tooth-age is demonstrated in Figure 4. Decrease of the a-lattice parameter in carbonate apatites is associated with an increase in carbonate content [1, 2, 17]. Higher number of planar carbonate ions substituting for the tetrahedral phosphate ions in the apatite structure is followed by an increased crystal structure disorder and reduction of the crystallinity as it is demonstrated by the broadening of the diffraction peaks in Figure 2. This is biologically important because the increase in carbonate content as a function of age also means an increase of the solubility of the dental apatite [6] and consequently the formation of calcium phosphate phases that alter the composition of the dental mineral. On the other hand, as Figure 5 shows, no significant changes of the c-lattice constant with the tooth-age were found with the exception of one sample. Accordingly, no significant substitution variations occur at the channel (hydroxyl) site as a function of the tooth-age.

**Figure 6:**
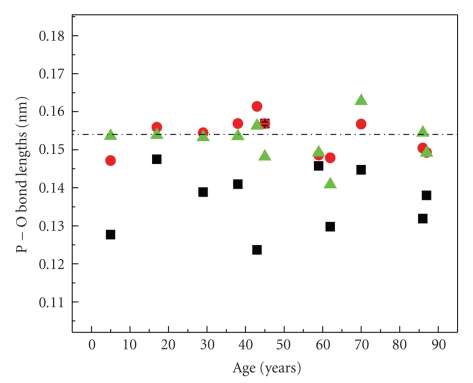
The refined interatomic distances between the atoms of the phosphate tetrahedron as a function of age. Squares mark the P-O1, triangles the P-O2 and circles the P-O3 bond lengths. Error bars are plotted for the P-O1 (not shown in this scale).

The refined interatomic distances between the atoms of the phosphate tetrahedron as calculated from the Rietveld refinements of the X-ray diffraction patterns are plotted in Figure 6 versus age. Notice that while in young-age teeth the tetrahedral distances P-O2 (triangles) and P-O3 (circles) are distributed around the ideal value of 1.54 Å (marked with the dashed line), they show disturbance in older-age teeth. Moreover, the P-O1 distances (squares) are noticeably disturbed in all samples compared to the P-O2 and P-O3 bond lengths. Distortion of the phosphate tetrahedron is correlated with the well-known lattice disorder caused by the carbonate for phosphate (B-type) substitution in natural and synthetic apatites, referred to as the “carbonate substitution problem” [18, 19]. Notice that this distortion is different from the one observed in carbonate natural fluorapatites and synthetic HAps that was studied earlier [17] in the sense that in those both the P-O1 and P-O2 interatomic distances of the atoms on the mirror plane of the phosphate tetrahedron were distorted by 3-4% because of the carbonate for phosphate substitution. Further investigation is required to draw conclusions on this subject regarding the dental carbonate HAp.

### 3.2. Thermogravitometric Analysis

**Figure 7:**
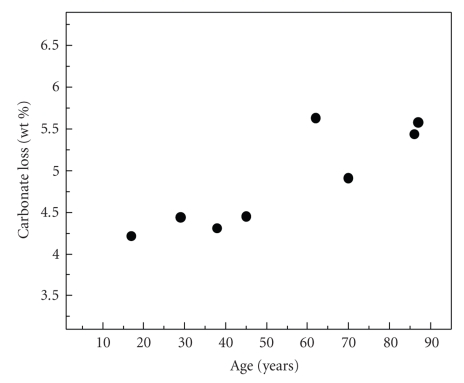
The wt% of carbonate loss of human dental apatite as a function age as evaluated from differential thermogravimetric analysis.

The wt% of the carbonate loss from several samples versus the tooth-age is plotted in Figure 7, as evaluated from differential thermogravimetric analysis in the temperature range above 600°C up to 950°C. Weight losses of absorbed, adsorbed water, or possible organic compounds that take place at temperatures less than 600°C were evaluated. An increase of carbonate content with the age is demonstrated in this figure confirming the correlation between the decrease of the a-lattice constant as a function of age of Figure 4 with an increased carbonate content.

### 3.3. FTIR Spectroscopy

**Figure 8:**
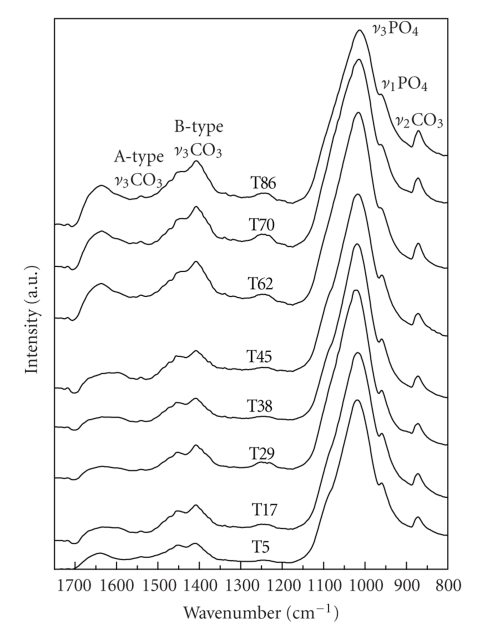
The 1750–800 cm^−1^ region of FTIR spectra of the human dental apatite measured from samples of 5 to 86 years old.

Figure 8 shows the 1750–800 cm^−1^ region of the FTIR spectra as collected from samples in the age range 5–86 years old. These spectra are characteristic of bio-apatites; the phosphate bands are identified by peaks at ~962 cm^−1^ (*ν*_1_ PO_4_ stretching IR mode), and the *ν*_3_ PO_4_ region which appears as a very strong broad asymmetric b and at ~1015 cm^−1^and consists of at least three submodes [20].

Strong peaks assigned to the B-type carbonate substitution (carbonate for phosphate ion) are observed at 872 cm^−1^(*ν*_2_ CO_3 _mode) and at 1405, 1450 cm^−1^(*ν*_3_ CO_3_). The weak bands in the *ν*_3_ CO_3 _region are attributed either to CO_3_^2−^ replacing PO_4_^3−^ ions without an adjacent OH^−^ ion [21] (at 1480 cm^−1^), or to the A-type carbonate substitution [2] (weak shoulders at 880 cm^−1^, ~1495 cm^−1^, and ~1530 cm^−1^). 

Organic phase related bands, mainly due to dentin, have been observed in the Raman spectra of tooth samples [22, 23]. In particular, the Raman bands peaked at 1250, 1450 and 1670 cm^−1^ were related with the amide III, amide II, and amide I bands, respectively. The amide bands have been observed in the IR spectra of tooth samples above 1500 cm^−1^ [24]. Therefore, the band at 1230 cm^−1^ band is attributed to amide III. The broad feature above 1600 cm^−1^ that consists of two subbands at 1610 and 1650 cm^−1^ can be attributed to overlapping bands of carbonate containing phases other than HAp (carbonate probably at the channel sites) [2], with amide III bands. The 1650 cm^−1^ band dominates over the 1600 cm^−1^ in the spectra from teeth older than 45 years old. Usually the higher-frequency subband is stronger at dentin untreated samples, while it loses intensity at enamel samples or upon treatment [24]. According to the aforementioned assignment the subbands behavior can be related either with the different content of dentin and enamel in the samples with age or with the secondary phases observed in the X-ray diffraction patterns. In favor of the secondary phases' explanation, some other weak bands at the *ν*_2_ CO_3_ region also imply the presence of carbonate in slightly different environments than A- and B-type as mentioned earlier for the weak bands in the *ν*_3_ CO_3_ region.

**Figure 9:**
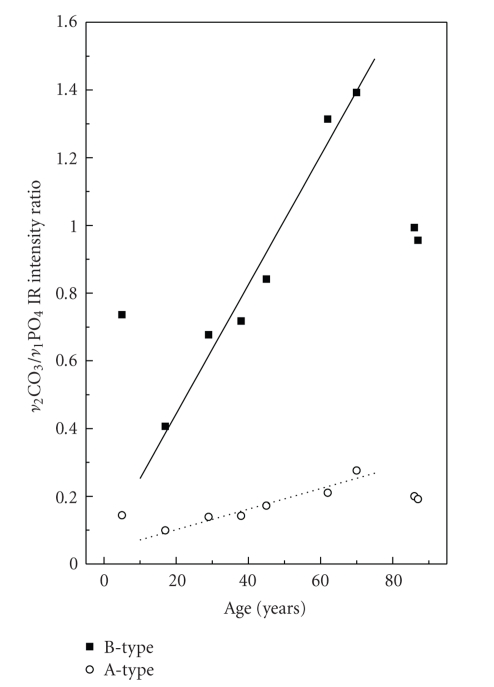
Ratios of the IR intensities of the *ν*_2[B]_ (880 cm^−1^) and *ν*_2[A]_ (870 cm^−1^) CO_3_ modes to the *ν*_1_ PO_4_ (960 cm^−1^) mode. Squares mark the B-type and open circles the A-type carbonate substitutions. An estimate of the relative carbonate content in human dental apatite as a function of the tooth-age is provided by these ratios. The lines are guides to the eyes.

In a previous work we have used the ATR technique for a quantitative estimation of the relative carbonate content in specimens of synthetic and natural carbonate apatites [20] from the ratios of the intensities of the *ν*_2_ CO_3_ modes to the *ν*_1_ PO_4_. Figure 9 presents the ratio of the IR intensities of the *ν*_2[B]_ CO_3_ mode to the *ν*_1_ PO_4_ (B-type carbonate substitution marked with squares) as a function of age. In the same figure the intensity ratios of the *ν*_2[A]_ CO_3 _to the *ν*_1_ PO_4_ mode (A-type carbonate substitution) as a function of age are marked with circles. Figure 9 clearly demonstrates a trend of increasing carbonate content with the tooth age, which is in a good agreement with the results of the XRD experiments shown in Figure 4 and the actual measurements of the carbonate loss of Figure 7.

The maximum B- to A-type relative carbonate content is approximately 5, a value that is close to other estimates in biological apatites [9, 15]. Other authors [9] have found similar results by using the relative intensities of the 1415 cm^−1^ (*ν*_3[B]_ CO_3_) to the 603 cm^−1^ (*ν*_4_ PO_4_) band and the 1545 cm^−1^ (*ν*_2[A]_ CO_3_) to the *ν*_4_ PO_4_ band, respectively. We prefer to use the ratios as in Figure 9 because there is no coexistence of more than one A- and B-type bands in the *ν*_2_ CO_3_ region as in the *ν*_3_, hence we avoid a possible fitting procedure uncertainty.

## 4. Conclusions

Consistent, systematic variations of average crystal structure properties of human dental apatite as a function of age were found in this study from XRD, TGA, and FTIR spectroscopy experiments. The decrease of the a-lattice constant versus age in dental apatite that is associated with increased carbonate content is related to increasing solubility which in turn results in a decrease of crystallinity and disturbance of the local lattice order of the biomineral. The approximately age-independent c-lattice parameter implies that the phosphate tetrahedron remains the main site of the carbonate substitution in the apatite lattice (B-type substitution) in the studied age-range. TGA measurements demonstrate increased carbonate content with the tooth age. FTIR spectra also show an increase of the B and A-type carbonate contents as a function of the age of the dental mineral phase with the B-type substitution up to 5 times greater than the A-type.

These trends of the average crystal structure properties of human dental apatite as a function of age could be useful in understanding the details of structural modifications in aging teeth. However, further research is required using specimens from a large, diverse pool in order to acquire statistical information considering that the tooth bioactivity is greatly affected by diet, diseases, or other local factors that consequently affect the evolution of the mineral phase in aging human teeth.
